# Multi-level determinants of vaccination of the American Indian and Alaska Native population: a comprehensive overview

**DOI:** 10.3389/fpubh.2025.1490286

**Published:** 2025-02-18

**Authors:** Junying Zhao, Rashmi Jaggad, Ying Zhang, Janis E. Campbell, Pallab K. Ghosh, James R. Kennedye, Tauqeer Ali

**Affiliations:** ^1^Department of Health Administration and Policy, University of Oklahoma Health Sciences, Oklahoma, OK, United States; ^2^Department of Biostatistics and Epidemiology, University of Oklahoma Health Sciences, Oklahoma, OK, United States; ^3^Strong Heart Study/Center for American Indian Health, Department of Biostatistics and Epidemiology, University of Oklahoma Health Sciences, Oklahoma, OK, United States; ^4^Department of Economics, University of Oklahoma, Norman, OK, United States; ^5^Chickasaw Nation Medical Center, Muscogee Creek Nation Medical Center, Department of Emergency Medicine, Kiowa Tribe of Oklahoma, Ada, OK, United States

**Keywords:** American Indian and Alaska native, safety, vaccination behavior, vaccination coverage, vaccination decision-making process, vaccine hesitancy, vaccine-preventable disease, Indian health service

## Abstract

**Context:**

American Indians and Alaska Natives (AIANs) are historically disadvantaged, losing 20 million (95%) of their population largely through epidemics since 1,520 and continuing lower overall vaccination coverage than other races. Determinants of this lower coverage are underexamined.

**Methods:**

Among peer-reviewed relevant articles since 1968, 39 studied AIANs solely; 47 drew general population samples, including AIANs. We employed rigorous economic definitions and framework of Individual Decision-Making Under Uncertainty. The Social-Ecological model identified determinants and mechanisms at five levels.

**Findings:**

Individual-level determinants include: (1) vaccine-preventable disease (VPD) and vaccine knowledge; (2) vaccine safety, efficacy, moral hazard beliefs; (3) preferences; (4) income and post-subsidy costs. Interpersonal-level determinants include others’ knowledge and preferences. Organizational-level characteristics of Indian Health Service, Tribal, Urban Indian (IHS/T/U) facilities include: (1) supply of vaccine products, providers, services; (2) provider cultural competency, vaccine recommendations, standing orders; (3) patient reminder/recall. Community-level characteristics include: (1) socioeconomics and geographics; (2) information infrastructure; (3) cultural values, practices, languages; (4) historical epidemic knowledge; (5) historical harms thus distrust in government, health system, science. Societal-level determinants include: (1) federal recognition and entitlements; (2) tribal self-determination; (3) state Medicaid enrollment; (4) structural racism.

**Policy recommendations:**

Tribal interventions may (1) increase AIANs’ knowledge about VPDs, vaccines, Medicaid enrollment; (2) design risk/cost–benefit calculations using scientific objective probabilities of vaccine safety and efficacy; (3) tailor messages to epidemic histories, narratives, values; (4) outreach by trusted messengers. I/T/U organizational interventions may reduce transportation costs while increasing provider supplies, cultural competency, and vaccine standing orders. Federal policies may increase IHS funding, tribal infrastructure, and AIAN data representativeness while eliminating structural racism and generational trauma.

**Conclusion:**

This article contributes to literature and practice. It is the first multidisciplinary, comprehensive overview of multi-level determinants and mechanisms of AIAN vaccination. Its findings highlight the gaps and limitations of laws and policies impacting AIAN vaccination. It recommends future research, culturally-appropriate interventions, and policies to close the gap to enhance AIAN vaccination and healing.

## 1 Introduction

Vaccines, one of the greatest inventions since the 18th century (1), have eradicated smallpox, significantly reduced prevalence of polio and measles, and saved approximately 8 million lives annually against these three vaccine-preventable diseases (VPDs) (2). However, the COVID-19 pandemic caused over 1.1 million deaths domestically (3), highlighting the complexities surrounding vaccine usage. The determinants of vaccine acceptance and refusal remain multifaceted and underexamined.

The American Indian and Alaska Native (AIAN) population is particularly and historically disadvantaged. AIANs lost an estimated 20 million people, 95% of its population, in epidemics brought by European settlers since the first smallpox case in 1520 (4). Smallpox deaths were largely reduced by implementing the Indian Vaccination Act of 1832 (5), and the disease was eventually eradicated in 1980 (4, 6). Tribal VPD experiences and vaccine successes have been passed through multigenerational narratives (7). However, the overall vaccination coverage of AIANs remains lower than that of other races in the United States (US). For example, the 2019–2020 influenza vaccination coverage among AIANs was 42.3%, significantly lower than the 52.8% of non-Hispanic whites (8). Forced relocation to reservations, broken treaties and promises, and underfunded Indian Health Service (IHS) have led to distrust in the federal government (9–12) and poor health, with 73 years of life expectancy at birth, 5.5 years lower than the US average (13, 14). Historical and sociocultural determinants uniquely influence AIAN vaccination behavior. Therefore, it is critical to understand the unique micro-to-macro determinants of AIAN vaccination behavior.

However, to date, little is known about this area. Literature focuses on individual-and community-level barriers for whites and other racial minorities. This article is the first comprehensive overview of the multi-level determinants of AIAN vaccination behavior from a multidisciplinary perspective, such as the income elasticity of demand for vaccines and moral hazard beliefs from economics. It draws attention to the gaps and limitations of laws and policies impacting AIAN vaccination. It recommends future research directions, local interventions, and broad government policies to address unique determinants and improve AIAN vaccination coverage, population health, and healing.

## 2 Materials and methods

We used various relevant search terms to search PubMed for peer-reviewed articles from 1968, when the literature was first available, to January 2024. Among 86 articles, 39 studied AIANs solely, and 47 drew general population samples, including AIANs. Evidence from general population samples always included AIANs unless otherwise specified; evidence from non-AIAN samples was occasionally mentioned to complete a logical argument.

**Figure 1:**
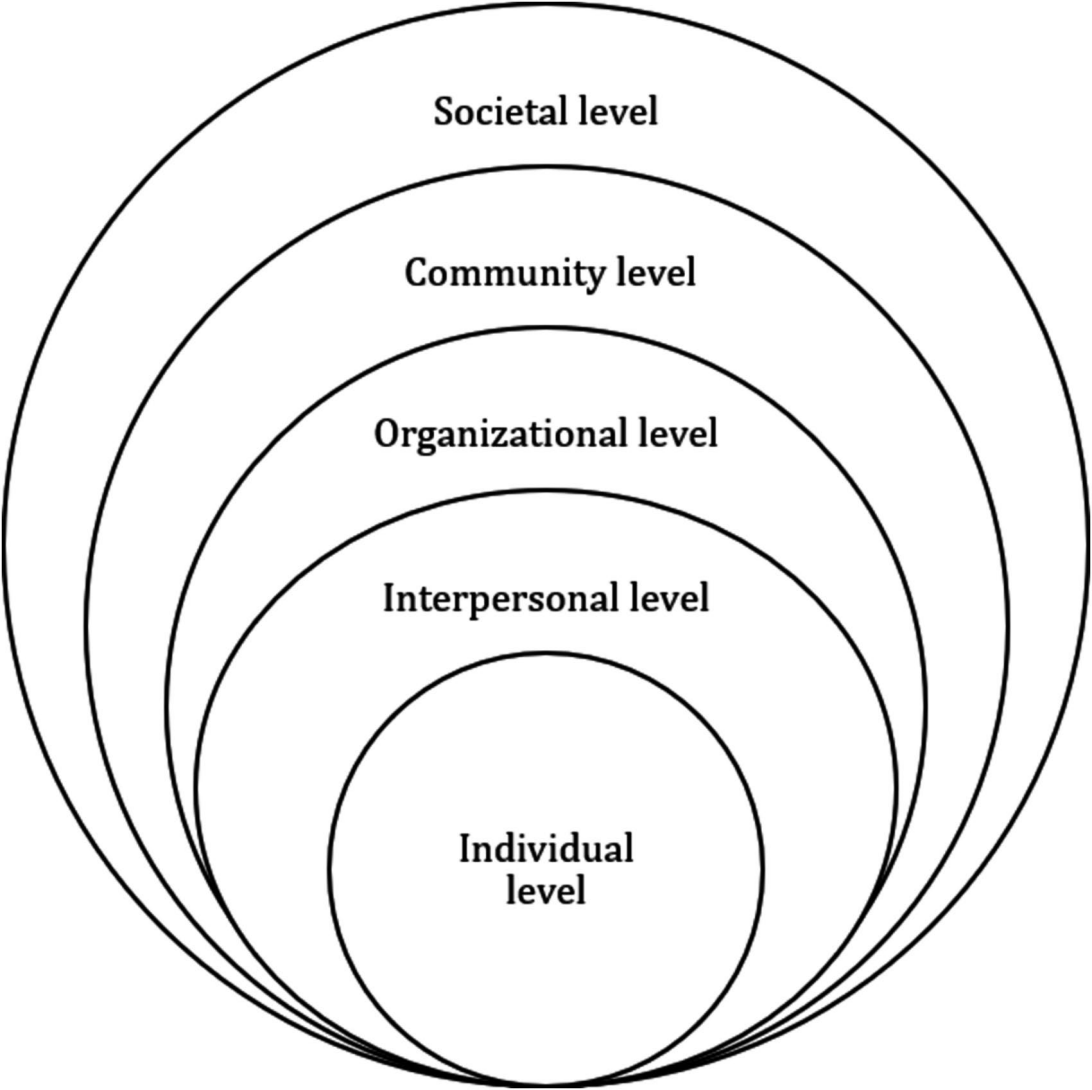
Diagram of Social Ecological Model (SEM). The social ecological model is a systems-level conceptual framework that considers how individuals and their environments interact to influence their behaviors (15). Source: Prepared by the authors.

The Social Ecological Model (SEM) is a systems-level conceptual framework that considers how individuals and their environments interact to influence their behaviors (15) (Figure 1). We applied the SEM to these articles and identified determinants and mechanisms of AIAN vaccination behavior at five levels: (1) individual, (2) interpersonal, (3) organizational, (4) community, and (5) societal level (Table 1).

**Table 1:** Determinants of vaccination behaviors of the AIAN population.

Levels of the social-ecological model	Determinants
Individual level	Knowledge Factual knowledge obtained from education Experience knowledge Beliefs Vaccine safety and efficacy beliefs; moral hazard beliefs Political beliefs Religious beliefs Individual Preferences: Preference over health Preference over health services Income affects the wealth and health status components of preference over vaccination Costs affects the wealth and health status components of preference over vaccination
Interpersonal level	Factual and experiential knowledge of interpersonal relationships Preferences of interpersonal relationships
Organizational level	Transport to health facilities Operational factors within health facilities Supply of vaccination providers Supply of vaccine products Supply of vaccination services Provider cultural competency Provider vaccine recommendations and standing orders Provider vaccine co-administration Patient vaccination reminder/recall School-based vaccination clinics
Community level	Socioeconomic status of AIAN communities Geographic location of AIAN communities Information infrastructure of AIAN communities Historical harms and resultant distrust in the federal government, health system, and science Historical and current experiential knowledge about VPDs and vaccines Cultural values, practices, and languages
Societal level	Federal level factors Federally unrecognized AIANs; Urban AIANs Public provisions of vaccine products and services for AIANs; Public subsidies to AIAN-eligible health insurance IHS underfunding challenge FDA pre-market vaccine clinical trials lack AIAN representativeness CDC and FDA post-market vaccine safety surveillance lack AIAN representativeness Tribal-level factors Tribal sovereignty, self-governance, and self-determination School immunization requirements for AIAN children State-level factor: Medicaid Manufacturers undersupply vaccines targeting virus genotypes highly prevalent among AIANs Researchers undersupply vaccine-related science on AIANs, lacking access to restricted data on AIAN race Structural racism leads to AIANs’ low overall vaccination coverage

Sources: Prepared by authors.

We applied multidisciplinary knowledge from economic and social sciences. Unlike vague terms (e.g., attitude, intention), preference is the theoretical foundation of economics and is mathematically defined (16). At the individual level, an individual preference is an ordering that compares choices A (e.g., to vaccinate) and B (e.g., not to vaccinate). Not preferring A over B means hesitating about A. After choosing an option (e.g., to vaccinate), it is uncertain whether an event (e.g., efficacy, adverse event) will occur, thus risk is present. Therefore, we apply the economic theoretical framework of Individual Decision-Making Under Uncertainty to vaccination decision-making (17). In it, the amount of risk depends on (1) the size of potential losses (costs) or gains (benefits) associated with the event and (2) the probability that the event will occur. The probability can be objective (frequentist) or subjective (perceived). Perceived probabilities of mutually exclusive events (e.g., adverse event exists or not) are beliefs, by definition (18). A preference ordering equals a set of utility functions that represent the preference (19). We used federal standard term “AIAN” rather than indigenous or Native American (20). The IHS uses a three-pronged system of IHS/Tribal/Urban Indian (I/T/U) facilities to provide health services to AIANs (21); we used these acronyms throughout the paper.

## 3 Individual level

### 3.1 Knowledge

#### 3.1.1 Factual knowledge obtained from education

Vaccination behavior is affected by knowledge acquired from or the ability to understand a proposition about VPDs, vaccines, or vaccination services (22). Lacking health or vaccine literacy impedes vaccination (23, 24). For example, over 57% of 31 surveyed IHS clinics in the Portland Area reported that patients lacked human papilloma virus (HPV) and vaccine knowledge (24). Convenience-sampled I/T/U providers reported that CDC Vaccine Information Statements (VISs) were “text heavy” for AIANs (25). Navajo traditional knowledge holders (TKHs) reported that hesitant members misunderstood vaccination for injecting actual viruses (26). Low literacy is attributed to low educational attainment. US adult national surveys found that, for each racial group, HPV and vaccine awareness decreased with low educational attainment (23).

The overall low educational attainment of AIANs has historical causes (27). Department of Interior (DOI) reported that, during 1819–1969, the federal Indian boarding school system was established to assimilate AIAN children (28). It comprised 408 schools across 37 states and over 1,000 other institutions (28). It forcibly removed AIAN children from their families, languages, religions, and cultures, resulting in physical and emotional abuse and, sometimes, deaths (29). DOI found that the US may have used monies held in tribal trust accounts to fund schools (28). Schools largely used manual labor, vocational skills, and militarized methods, including corporal punishment; at least 19 schools accounted for over 500 AIAN child deaths (28).

The system left AIANs with adverse childhood experiences and generational trauma. Many dropped out of schools (30), lost their languages and identities, withdrew from white and tribal worlds (31), and developed mental illnesses (27, 32). Their fear discouraged their offspring from attending institutionalized education and continued generational trauma (33). For example, in 2014, the AIAN high school graduation rate of 67% and, particularly, the DOI-Bureau of Indian Education (BIE) school graduation rate of 53% were lower than the 80% national average; in addition, the AIAN youth suicide rate was 2.5 times the national average, the second leading cause of youth death (27). In 2018, BIE schools still did not require teachers to have knowledge about AIANs except state certifications, putting their cultural incompetence in question for educating AIAN students (31).

Interventions may (1) develop pictorial VISs in tribal languages; (2) educate AIANs on VPDs and vaccines, as non-dangerous fragments of viruses, in I/T/U facilities, tribal schools, and communities; (3) use the IHS boarding school toolkit to heal generational trauma (34); (4) improve graduation rates and educator cultural competency in BIE-funded schools.

#### 3.1.2 Experiential knowledge

Negative experiences impede vaccination, such as culturally incompetent providers (35–38). Interventions may strengthen culturally competent communication and uneventful delivery, including adequate consent, painless vaccination, and timely responses to adverse events following guidelines (39).

### 3.2 Beliefs

#### 3.2.1 Vaccine safety and efficacy beliefs; moral hazard beliefs

Recall that beliefs are perceived probabilities of vaccine efficacy, adverse events, or other unexpected consequences. The amount of a perceived risk includes both losses (or gains) and associated perceived probabilities; multiplying both produces the amount of perceived costs (or benefits). AIAN parental concerns or perceived risks of child HPV vaccination were reported by convenience-sampled I/T/U providers (25). The first concern was efficacy: Parents were unsure if the vaccine would work well, that is, low perceived benefit, as a product of perceived probability and size of protective immunity. The second concern was about safety: Parents were worried about the chance and severity of side effects, that is, high perceived cost, as a product of perceived probability and severity of adverse events. The third concern was moral hazard: Parents believed that children would engage in risky sexual behaviors, thinking the HPV vaccine would prevent them from sexually transmitted diseases (STDs). Such parental perceived probability and severity of child STDs incurred additional perceived costs of vaccination that outweigh the perceived benefits. The resulting perceived risk rendered parents hesitant (40). Similarly, nearly half of Arizona tribal participants in an educational intervention by community health representatives (CHRs) expressed safety and efficacy concerns about COVID-19 vaccines (41). Also, Navajo TKHs reported a lack of intellectual explanation about COVID-19 vaccine risks (26).

Interventions should (1) extract numerical measures of objective probabilities from scientific literature and FDA clinical trials, (2) deliberate objective probabilities among AIANs, and (3) examine how much AIANs update their subjective probabilities (beliefs) and thus change their vaccination behaviors.

#### 3.2.2 Political beliefs

Individual normative statements about how governments should handle an issue can affect their vaccination (19). Among convenience-sampled American adults, self-identified Republicans and Independents were more COVID-19 vaccine-hesitant than Democrats (30, 42, 43). Convenience-sampled American and Canadian adults reported beliefs in the political spectrum: (1) communism left-wing or socialism; (2) liberal; (3) center or moderate; (4) conservative; (5) fascism right-wing or authoritarianism. Among the 48 indigenous participants in the sample, the liberal, center, and conservative accounts for approximately one third each. AIANs were more COVID-19 vaccine-hesitant than all racial groups except African Americans. Given a left-wing, conservative, or right-wing belief, a cross-racial group difference in vaccine hesitancy existed (44). However, these results neither attributed political beliefs to hesitancy nor reported, given a racial group, a cross-political-belief group difference in hesitancy. Research may address how (e.g., framing, agenda setting) political beliefs affect vaccination (30). Interventions may address pragmatic (e.g., safety) concerns while respecting political beliefs.

#### 3.2.3 Religious beliefs

Protected by Title VII of the Civil Rights Act of 1964, religious beliefs are subjective probabilities of something divine and associated normative statements about how one should behave in relation to the divine (45–47). The traditional religious dimension of Indian life, that had previously caused fragmentation among certain AIANs (Indian traditionalist vs. Indian Christians), was an important aspect of tribal identity (214, 215). For example, spirituality influences COVID-19 vaccine uptake, including acts that honored spirit and maintaining positivity and harmony through traditional knowledge (e.g., connect viruses to creation stories), traditional practices (e.g., songs, prayers, ceremonies), and traditional medicine. 26 For another example, I/T/U providers reported AIAN parental opposition to child HPV vaccination due to religious or moral reasons (25). Research may address how religious beliefs affect vaccination.

### 3.3 Preferences

#### 3.3.1 Preference over health

Unhealthy behaviors reveal a low preference for health (40, 48–50). For example, smokers were more COVID-19 vaccine hesitant than non-smokers (49). Also, low health status may motivate a high preference for health. For instance, cancer survivors are at high risk of VPDs and prefer COVID-19 vaccination (51). AIANs preferred natural immunity most among all racial groups (44).

Interventions may educate individuals with unhealthy behaviors, high risks of VPDs, or preferences for natural over vaccine-induced immunity on two aspects: (1) similar B-cell biological mechanism of both immunities; (2) different risks with actual virus infection and high mortality for natural immunity, but killed or weakened virus and low mortality for vaccine-induced immunity (52).

#### 3.3.2 Preference over health services

Vaccination behavior can be affected by preferences over the type of medicine and the frequency, specialty, and setting of provider visits. For example, some AIANs prefer traditional over Western medicine (26). For another example, five-year Medicare survivors of colorectal cancer reported retrospective findings: (1) Asian and Pacific Islanders (APIs) who visited physicians six times or more had higher odds of influenza and pneumococcal vaccinations than those who visited less; (2) Those who visited primary care physicians (PCPs) only and both PCPs and oncologists had higher odds of both vaccinations than those who visited other specialists (53). Randomly sampled Colorado households reported parental preference ordering of adolescent vaccination settings, starting with the most preferred: family physician or pediatrician office, public health clinic, school health clinic, emergency department, and obstetrics and gynecology clinic, rather than retail and family planning clinics (54). Particularly, a randomized controlled trial intervention in an emergency room among general pediatric patients offered influenza vaccines as treatment in addition to vaccine education. It increased vaccination rates in treated patients and accompanying families (55).

Interventions may elicit AIAN-specific preferences (1) over traditional and Western medicine, then explore integrated approaches for vaccination, (2) over the frequency, specialty, and setting of providers and I/T/U facilities, then incentivize the most preferred providers and facilities to recommend routine vaccines to AIAN patients.

#### 3.3.3 Income affects the wealth and health status components of preference over vaccination

Recall that preference is represented by utility functions whose main inputs are wealth and health status. Earned income each time adds to accumulated wealth over time, directly increasing utility. Disposable income sets a budget for consuming goods (e.g., vaccine products) and services (e.g., vaccination services) that improve health status; thus income indirectly increases utility. Therefore, income affects preference over vaccination.

The overall low-income level of AIANs persists, despite some tribes are richer than general US populations due to good investments, casinos or oil fields on their tribal lands. In 2019, AIANs had a median household income of $49,906, a 20.3% poverty rate, and a 7.9% overall unemployment rate, worse than the $71,664, 9.0, and 3.7% for non-Hispanic whites, respectively (56).

Income affects vaccination through three potential mechanisms: (1) the slope and (2) the acceleration of the income-vaccination relationship, given vaccination as consumption; (3) vaccination as investment. First, low-income AIAN women in Wisconsin had vaccine hesitancy in preference, thus vaccination delay and non-adherence in behavior (57) possibly due to the inability to pay. Second, income elasticities of demand for vaccination, as a consumption, measure if income changes, how much demand for vaccination will change. We hypothesize that if poor individuals’ income decreases by one unit, their demand for vaccination decreases by more than one unit. Yet, no research has estimated this measure or tested this hypothesis. Third, we hypothesize that low-income individuals have low budgets and thus prioritize primary goods consumption (e.g., food, shelter) over investment. Vaccination, as an investment, reduces future VPD-related health losses and associated financial losses (e.g., fewer medical bills) while generating future productivity gains and associated financial returns (e.g., more wage income). Yet, no research has tested this hypothesis. Finally, employment status affects wage income, thus vaccine consumption. Having a full-time job facilitates vaccination (58).

Research should measure income elasticities of demand for vaccination and test vaccination as an investment decision. Interventions may enhance job training and opportunities for low-income or unemployed AIANs.

#### 3.3.4 Costs affect the wealth and health status components of preference over vaccination

Health service costs decrease wealth, generating disutility, but health services (e.g., vaccination) increase health status, producing utility. If disutility outweighed utility, an individual would prefer no vaccination; that is, high costs impede vaccination (37, 59, 60). Costs include time and transportation to facilities (section 5.5.1), and real prices of vaccination services after cost reductions by public provision or subsidy (e.g., Vaccines for Children (VFC), IHS, Medicaid, sections 7.1.2–7.1.3) or by private insurance, such as employer-based health insurance. Moreover, low-income individuals generally had high price elasticities of demand for health care (61); if the price increases a little, their demand decreases substantially. Yet, no research has estimated this measure for vaccination.

Large employers must offer minimum health insurance coverage to full-time employees by law (62, 63). However, AIANs had the highest unemployment rate among all racial groups at 7.9 and 6.2% in 2019 and 2022, respectively (64). Among randomly sampled US adults, unemployed individuals were 30% less likely to be COVID-19 vaccinated (58). In the US Census, parents whose daughters initiated HPV vaccination were more likely to be full-time employed (50).

Taken together, future research should first measure the general and AIAN-specific (1) preferences regarding risk, including risk-averse, neutral, and loving (17), and (2) income and price elasticities of demand for vaccination. Interventions may then educate individuals on (1) vaccination as an investment decision and (2) the vaccination decision-making process under uncertainty: It calculates the perceived risk of vaccination, which aggregates perceived costs and benefits as losses and gains with associated perceived probabilities (beliefs), and leaves each individual to decide based on their own perceived risk and risk preference. Policies can then target individuals whose vaccination demand is highly sensitive to real price or income. Policies should address transportation and financial barriers to AIAN vaccination: IHS underfunding (section 7.1.3), Medicaid low enrollment (section 7.1.2), and unemployment.

## 4 Interpersonal level

### 4.1 Factual and experiential knowledge of interpersonal relationships

Interpersonal relationships can be sources of vaccine factual and experiential knowledge. Parental unawareness of VPDs or vaccines hinders child vaccination (65). Racial minority parents of HPV-unvaccinated children in Los Angeles County preferred trusted relationships to navigate vaccine information (66). Similarly, an educational intervention in Arizona tribes found that healthcare professionals (HCPs) and CHRs were the most trusted health messengers for COVID-19 information, whereas social media contacts were among the least trusted (41). Experiences with HCPs impacted minority parental trust in HCP vaccine recommendations for children (66). Negative vaccination experiences of others also discouraged some Navajos from vaccinating (26).

Interventions may educate interpersonal relationships who lack knowledge about VPDs, vaccines, or vaccination services, such as peers, families, and accompany family members in schools, households, and I/T/U clinical settings. Interventions may also identify financial resources to incentivize HCPs for routine vaccine recommendation and patient satisfaction.

### 4.2 Preferences of interpersonal relationships

Preferences of others can influence AIAN vaccination. For example, parents preferred no child HPV vaccination due to beliefs in unintended consequences (e.g., moral hazard) (65) (section 3.2.1). Navajo TKHs preferred COVID-19 vaccination due to largely perceived benefits from the improved health of their own and family, especially grandchildren (26). Preferences of and recommendations by vaccinated elders, TKHs (26), or other trusted community members (67) increased an AIAN’s likelihood of COVID-19 vaccination.

Triadic relationship-based intervention had HCPs provide parent–child HPV vaccine and sex education and address their concerns separately and effectively (65). Some states require parental consent for child vaccination, while others do not for children over certain ages (23, 24). Similar interventions may educate hesitant relationships on knowledge about and preferences for routine vaccines, and incentivize trusted community members and traditional HCPs to recommend routine vaccines.

## 5 Organizational level

### 5.1 Transportation to health facilities

Transportation barriers include remote locations of the nearest I/T/U facilities (43) (organizational-level determinant), lack of private (individual-level) or public (societal-level) transportation tools (66, 68), and expensive transportation costs (e.g., time, gas). A pneumococcal vaccination multi-intervention during 2001–2007 targeted high-risk IHS patients in Arizona. It provided vaccinations in community, home, inpatient, and outpatient settings with standing orders, electronic records, electronic and printed reminders, and staff and patient education. It effectively achieved the corresponding coverage goal in Healthy People 2010 (69). Other I/T/U facilities may implement similar multi-interventions for routine vaccines.

### 5.2 Operational factors within health facilities

#### 5.2.1 Supply of vaccination providers

The longstanding underfunding of the IHS (section 7.1.3) has led to understaffing and under-serving consequences. We found that IHS spending on public health nurses decreased from $14.063 million in fiscal year (FY) 2016 to $13.709 million in FY2017, both in FY2016 dollars after adjusting for inflation (70). The US Government Accountability Office (GAO) found that the 2018 IHS vacancy rates of HCPs and physicians averaged 25 and 29%, respectively (71). Most IHS-designated shortage areas of primary care were isolated hardship posts. Reasons for the IHS labor shortage included the lack of funding to offer competitive salaries and pay raises, and potential pay delays due to federal government shutdowns (72).

The GAO and Congressional Research Service recommended Congress authorize IHS advance appropriations to avoid recruitment interruptions (72, 73). The American Medical Association recommended IHS partner with academic medical centers, utilize their trainees, and offer competitive compensation (74). We recommend policies to address the IHS underfunding challenge (section 7.3). To enhance the immunization workforce, we recommend IHS facilities expand education and outreach services of CHRs from COVID-19 vaccines (75) to all routine vaccines, and state governments expand Medicaid coverage of community health worker (CHW) services (75) to CHR services for AIAN Medicaid enrollees. CHRs are trusted tribal members (41) and trained by the IHS CHR Program to provide health education and promotion, coordination, and translation (75). CHR activities are managed by tribal governments through legal arrangements (e.g., contracts and grants) with the IHS (76). CHRs assess IHS and non-IHS health resources in tribes, facilitate resource utilization, and assist IHS and non-IHS health agencies in (re)designing services for community needs (76, 77).

#### 5.2.2 Supply of vaccine products

Vaccine supply issues for AIANs vary between pandemic and routine vaccines and across federal, state, and tribal entities. The National Academy of Medicine identified the 2009 H1N1 influenza vaccine distribution issues (78). First, states varied distribution channels either directly or indirectly through local health departments or clinics to tribal populations (78). Second, federal distribution sometimes affected state distribution; delayed communications about IHS employee vaccines complicated Alaska state vaccination plans that included IHS facilities (78). Third, IHS employee vaccines and tribal population vaccines were sometimes difficult to distinguish in small IHS facilities, because the former came from the federal government and the latter from jurisdictions (78). Fourth, tribal elders were excluded from CDC-recommended age groups, including all aged 6 months-24 years and high-risk individuals aged 25–64 years (78, 79).

The COVID-19 vaccine distribution had relatively fewer issues in a de-centralized and multi-phase way: (1) Estimates of doses needed at I/T/U facilities in the early allocation phase were collected by Area Vaccine Point of Contacts (AVPOCs) and submitted to the IHS-National Supply Service Center (NSSC) located in Oklahoma City; electronic orders can be made by facilities as needed to NSSC in later phases; (2) NSSC reviewed and adjusted estimates or orders, and submitted to CDC; (3) CDC distributed doses to I/T/U facilities either indirectly through contracted wholesale distributors or directly through manufacturers for ultra-cold vaccines (80). Moreover, certain state governments (e.g., Alaska) distributed vaccines to T facilities (81). In a GAO performance audit of IHS, most hospitals expressed high confidence about sufficient supplies of vaccine-related items (e.g., syringes). However, more than half of 24 surveyed IHS hospitals encountered challenges to provide space and staff to manage and administer vaccines and provide IT systems to track administered doses; almost all of them faced patient vaccine hesitancy (82).

Routine (e.g., influenza) vaccines are also efficiently distributed through the de-centralized NSSC system based on orders from each I/T/U facility under the NSSC Annual Purchase Program (83). Vaccine supply targeted racial and ethnic minority groups such as AIANs may reduce VPD disparities in AIAN and risk among the general population (84). Research should identify whether, when, and why a pandemic or routine vaccine shortage might exist in I/T/U facilities to inform policy solutions. Efficient communication and coordination are needed among IHS and other (e.g., CDC, state, tribal) entities that distribute, receive, or administer vaccines from different sources.

#### 5.2.3 Supply of vaccination services

Vaccine products and providers produce vaccination services. We found that the total spending on public health by the IHS was $155.734 million in FY2016 but decreased to $152.043 million in FY2017, both in FY2016 dollars (70). Lack of funding impeded the IHS supply of vaccination services to AIAN patients. For example, the HPV vaccine Gardasil 9 is priced at $178.33–$287.54 per dose (85). Some IHS clinics did not provide it to AIAN female patients aged 19–26 years because no funding was available at the IHS internally, from insurers with ambiguous reimbursement policies, or from patient’s out of pocket (24). GAO may investigate insurance compliance with the Affordable Care Act (ACA) no cost-sharing requirement for routine vaccines. In November 2022, the IHS directed all facilities to implement an E3 policy: “Every patient at every encounter should be offered every recommended vaccine when appropriate” (86). Performance measures should be reported.

#### 5.2.4 Provider cultural competency

Culturally competent provider communications may facilitate AIAN patient trust in provider vaccination recommendations [section 6.6 (87, 88)]. In an educational intervention in three Arizona tribes, the majority of tribal participants felt the COVID-19 vaccine education given by trusted CHRs was culturally and locally relevant (41). Cultural competency interventions in vaccination providers were not found but suggested in the literature (89). Interventions in dental aids provided culturally appropriate education and routine dental services to Alaska rural residents and effectively enhanced their access (90). We suggest similar training for I/T/U vaccination providers.

#### 5.2.5 Provider vaccination recommendations and standing orders

Where authorized by a facility medical director or state law, a standing order enables a non-physician HCP to assess and vaccinate persons who meet specific CDC criteria without a physician order (91–93). State laws vary in authorizing which non-physician HCPs should administer which vaccines to which permissible patient populations in which settings (93). However, I/T/U facilities are not subject to state laws (94). Standing orders, together with other interventions, effectively increased pneumococcal vaccination coverage among high-risk patients in IHS facilities in Arizona (69). Provider in 16 I/T/U facilities in five IHS regions (Great Plains, Nashville, Navajo, Oklahoma, Portland) reported that most facilities had HPV vaccine standing orders in place, but some lacked consistent implementation (95). Randomly sampled parents of AIAN adolescents who accessed T facilities in Oklahoma found that the prevalence of adolescent HPV vaccination whose parents received HCP recommendation was higher than those whose parents received none (96). A similar effectiveness of HCP recommendation in HPV vaccine initiation was found in a national survey (50). Interventions at I/T/U facilities may increase implementation consistency of standing orders and recommendations for other routine vaccines.

#### 5.2.6 Provider vaccine co-administration

Co-administering multiple vaccines at different injection sites in a single visit saves patients time and may increase the overall coverage. Co-administering HPV and meningococcal vaccines may particularly reduce stigma and increase HPV vaccination (96). Interventions may implement co-administration at I/T/U facilities, following CDC best practice guidelines for immunization (97).

#### 5.2.7 Patient vaccination reminder/recall

Patient reminder/recall facilitates vaccination. Convenience sampled I/T/U providers reported that 69% of them used electronic health records (EHRs) or registries to track patient HPV immunization schedules; more than 50% of them requested patients to schedule the next appointment upon receiving the first dose (25). The IHS uses an EHR, “Resource Patient Management System” (RPMS), with an immunization component. The RPMS generates provider and patient reminders/recalls and monitors community-level vaccination coverage. Providers also used paper-based cards, mailing letters, and postcards (25). Parent focus groups of AIAN adolescents in T facilities shared that automated text reminders would help increase HPV vaccination (98). A quasi-experiment gave parents of non-AIAN children in private pediatric practice the intervention of HPV vaccine educational brochure and telephone reminders prompted by electronic alerts. Intervened parents were more likely than non-intervened parents to have their children initiate and complete the HPV vaccine series (99). Interventions may increase using EHR immunization reminder/recall functions and other follow-ups in all I/T/U facilities (65).

### 5.3 School-based vaccination clinics

BIE and tribally controlled schools partner with I/T facilities to provide on-site care. IHS facilities connected vaccination personnel to these schools and hosted COVID-19 vaccination events for faculty, staff, and students (100). Four focus groups were conducted among women in communities and HCPs in I/T facilities in the IHS Aberdeen Area; they suggested school-based HPV vaccine clinics and community-wide education but recognized the limited school capacity to do so (101). Native American Health Center, a community health center in San Francisco, had its school-based health centers provide services (e.g., immunizations) in three school districts. The Los Angeles County Department of Public Health asked nearly 3,000 schools to host COVID-19 vaccine clinics for students, families, and communities while providing logistics guidance and tools (102). I/T facilities may coordinate and complement routine vaccination events at BIE and tribally controlled schools.

## 6 Community level

### 6.1 Socioeconomic status of AIAN communities

Economic resources are closely associated with the wealth of an individual or community (103) and impact their vaccination. Low socioeconomic communities have fewer health facilities and services (35, 49). National surveillance showed a positive association between census tract income level and influenza vaccination coverage among influenza-related hospitalized patients aged 65 years and older, ranging coverage from 35% in the poorest tracts to 48% in the richest (104). Anti-poverty and poverty-based Medicaid policies were introduced to the general population and had mixed results on immunization (section 7.1.2). Interventions to enhance immunization may use surveillance data to identify AIAN communities in high-poverty census tracts (104).

### 6.2 Geographic location of AIAN communities

Rurality was associated with low likelihood of COVID-19 vaccination nationwide (105). COVID-19 vaccination outreach varied (68). T facilities in Alaska delivered vaccines by water taxi, sled, and plane and administered vaccines on tarmacs and during home visits. The Pueblos partnered with the New Mexico Government to distribute vaccines to reservations and with tribal Emergency Medical Services to administer vaccines at drive-through sites. A tribal community organization in Oregon partnered with rural public transportation providers (106) to transport members to vaccine clinics at tribal longhouses (107). Community-based interventions may involve community stakeholders to implement and expand immunization programs, hold mobile clinics in tribal complexes, and expand CHR outreach from COVID-19 to routine vaccines.

### 6.3 Information infrastructure of AIAN communities

Digital technologies are one source of vaccine information. The digital divide refers to unequal levels of digital literacy and access to digital technologies (108). In 2020, 18% of people living on tribal lands could not access broadband internet, higher than the 4% in non-tribal areas (109, 110). According to the GAO (111), tribes lacked the financial resources to afford the high costs of high-speed internet access and lacked administrative and technical expertise in such access. The Federal Communications Commission (FCC) and the Department of Agriculture lacked coordination to develop joint outreach and training to increase such access on tribal lands. The FCC lacked performance goals and measures to implement its National Broadband Plan to households and to fund libraries and schools through the E-rate program on tribal lands (111). Lacking internet access as a barrier to COVID-19 vaccination was discussed and evidenced (112) in quantitative and qualitative studies for Navajos (113), urban AIANs (59, 114), and indigenous people in Canada (115, 116). However, due to vaccine misinformation and disinformation, digital access is a double-edged sword. Interventions may improve the digital and vaccine literacy of community members. Policies may address tribal digital infrastructure issues and expand digital access from libraries and schools to communities (110).

### 6.4 Historical harms and distrust in the federal government, health system, and science

AIAN tribes signed treaties that ceded their title to lands to the federal government in exchange for protection, health services, and cash while moving to reservation lands. “The federal government holds title to the land in trust on behalf of the tribe” (117). That is, the federal government, as the trustee, is legally obligated to hold the land in a manner that is in the best interest of the tribe as the beneficiary. Treaties were made with the understanding that treaties would be in place for “as long as the sun shines, the grass grows, and the river flows” (118–120). However, the General Allotment Act of 1887 “authorized the President to break up reservation land into allotments to be parceled out to individuals” (121). “The title to the parcel stayed in trust, but for the individual Indian, not the tribe. Allotted land…can be sold [and] condemned” (122). Tribes perceived the allotment law as not in their best interest and amplified their distrust in the federal government (90). For example, in *Lone Wolf v. Hitchcock* (1903) (123), the Kiowa Tribe Chief Lone Wolf sued the DOI Secretary to halt the allotment of the Kiowa, Comanche, and Apache Reservation in Oklahoma, as delineated in the Medicine Lodge Treaty of 1867. The US Supreme Court held that although the US Constitution reflected in its declaration that Indian treaties are the “supreme Law of the Land” (124), the US Congress has “plenary power” to unilaterally abrogate a treaty in whole or in part when circumstances change or the national interest demands (118). This distrust among AIAN people in the federal government reverberates today. Concerns about management issues during the COVID-19 pandemic have worsened the distrust (44).

AIAN members also experienced misconduct in certain IHS facilities and distrusted the health system. The GAO investigated 3,406 sterilizations of AIAN women aged 15–44 performed during FY1973-1976 in IHS facilities in four out of 12 IHS service areas (Aberdeen, Albuquerque, Oklahoma City, and Phoenix). The GAO found “no evidence of IHS sterilizing Indians without a patient consent form on file, [it] did find several weaknesses in complying with the [Department of Health and Human Services, DHHS] regulations … (1) sterilization of persons under 21 years of age, (2) inadequately documenting what the subjects were told before signing the form, (3) lack of physician understanding of regulations, and (4) lack of definitive requirement for consent at contract facilities” (125). The misconduct has been told through intergenerational narratives (126, 127) and led to community distrust in the recent federal HPV vaccine recommendation, resulting in low HPV vaccination coverage (50).

AIAN communities also experienced misconduct and distrust in research (126). In *Havasupai Tribe v. Arizona Board of Regents* (2004), the Tribe sued the Board for misusing their DNA data beyond the consented study and settled for $700,000 and returned blood samples (126, 128). However, AIANs were “willing to trust science if it was presented by community members” (67).

Distrust resulting from land issues may be healed in the context of health care if the federal government appropriates sufficient funds for the IHS to meet AIAN needs. Credible sources (41) and communication transparency help address distrust, such as communicating why some communities were supplied AstraZeneca vaccines because they had no ultra-cold capacity for Pfizer vaccines (114).

Informed consent policies may be revisited. The CDC provides VISs without consent requirements for routine vaccines (129). States might require immunization-related consent from Medicaid enrollees (130). The IHS requires all its providers to obtain patient consent before treatment but does not specify patient immunization consent (131).

Research must comply with up-to-date laws, policies, and IRB processes prohibiting misconduct. Researchers must learn community history and culture, and engage and resolve issues early (128).

### 6.5 Historical and current experiential knowledge about VPDs and vaccines

AIANs had lost about 20 million people, 95% of its population, in historical epidemics since first reported in 1520: bubonic plague, chickenpox, cholera, diphtheria, influenza, malaria, measles, pertussis, smallpox, STDs, tuberculosis, and typhoid (4). Smallpox deaths had been largely reduced by vaccines since the first administration to AIANs in 1832 and were eventually eradicated in 1980 (4, 132). Tribal deaths from VPDs and successes of vaccines were passed through multigenerational narratives to younger generations (7) as evidenced by AIAN TKHs (26) and elders (36) who supported COVID-19 vaccines. Interventions may educate members on elders’ narratives and tribal histories with epidemics.

### 6.6 Cultural values, practices, and languages

AIAN cultural values facilitate vaccination, such as respect for elders and “community first.” AIANs preferred COVID-19 vaccinations to protect elders and the community, thus preserving their culture (87). Tribal languages are cultural heritage. TKHs often speak tribal languages and practice herbal medicine, prayers, chants, and ceremonies for members to address their health issues. TKHs could help name a virus or vaccination in tribal languages, connect the virus to tribal creation stories, help members understand where the virus comes from, and reduce fear of vaccination while “maintaining cultural respect for all living beings, including the virus” (26). Some TKHs combined practices from both traditional and Western medicine. These approaches could collectively facilitate vaccination among members (26). For example, in the Kiowa Tribe of Oklahoma, vaccination was named to be pierced with medicine, “Doi Tawt Gaw,” where “Doi” means medicine and “Tawt gaw” means to be stabbed or pierced (133). Laura D. Pedrick (“Tone-Adle-Mah,” Lame Leg Woman), Kiowa member and field matron, was born on the reservation during the US Civil War and one of the first persons to administer smallpox vaccines to tribal members in Oklahoma during the 1890s-1900s (134–137). Interventions may (1) consult TKHs about cultural appropriateness; (2) tailor materials in tribal languages and short texts, audios, or videos and contain native songs, personal narratives, and cultural symbols and values (138); (3) display tailored materials in I/T/U clinical settings and multimedia platforms in community settings.

## 7 Societal level

### 7.1 Federal-level factors

#### 7.1.1 Federally unrecognized AIANs; urban AIANs

The 2020 Census estimated 3.7 million self-identified AIANs alone and 5.9 million additional AIANs and another race, totaling 9.7 million, 2.9% of the US population (139). Congress terminated 109 tribes during the 1950s-1970s (126, 140). The DOI applies the Federally Recognized Indian Tribe List Act of 1994 (141, 142), and has recognized 574 tribes as of 2022 (139). The DHHS-IHS only serves 1.6 million AIANs living near reservations as part of the approximately 2.6 million members of recognized tribes (143). Unrecognized tribes share historical traumas and health risks with recognized tribes. However, they are not captured in health literature (144) or eligible for I/T services and public insurance (145, 146) but for cost-sharing services at U facilities (147). Moreover, about 60% of AIANs live in metropolitan areas (139) and have to travel to I/T facilities, mostly located on or near reservations, or seek cost-sharing services at U facilities with only about 1% of IHS annual appropriations (148). Congress may consider reducing time and financial barriers to the federal recognition process and increasing U facility appropriations.

#### 7.1.2 Public provisions of vaccine products and services for AIANs; public subsidies to AIAN-eligible health insurance

Having public provisions (e.g., VFC) for healthcare goods (e.g., vaccine products) or services (e.g., IHS, section 7.1.3) or having public subsidies for health insurance (e.g., Medicaid) facilitates AIAN vaccination (149–152). VFC is a federal entitlement provision program, a right granted by Section 1928 of the Social Security Act of 1993 (153) for AIAN, Medicaid enrolled, uninsured, and underinsured children. The VFC provides free vaccines for providers serving eligible children but pays no vaccine administration fees at $10–25 nationwide (154), a financial barrier unless covered by insurance, such as Medicaid (155, 156).

Medicaid is a federal entitlement insurance program for low-income individuals (145), with usually 100% federal matching rates for AIAN enrollees (157). Medicaid reimburses IHS services to AIAN enrollees (90). Although AIANs had the highest poverty rate amongst all racial groups (158), they had the least likelihood of accessing Medicaid, lacking knowledge about Medicaid or its application process (90). Moreover, the ACA was passed in 2010 and implemented in 2014. It required most insurers to impose no cost-sharing for preventive services (159), increasing HPV vaccination among females (160). The ACA also expanded Medicaid eligibility for more relatively poor individuals. AIAN uninsured rates decreased from 29 and 24% in 2004 and 2013 to 21 and 14.9% in 2014 and 2019, continuously lower than non-Hispanic whites at 6.3% in 2019 (90, 139). Medicaid expansion had mixed results on HPV and influenza vaccinations (149–152, 160–164).

Interventions may increase Medicaid insurance literacy and assist applications among AIANs in community and I/T/U clinical settings, which will help address the IHS underfunding challenge. GAO may investigate insurance compliance with the ACA requirement of no immunization cost sharing, including administration fees.

#### 7.1.3 IHS underfunding challenge

IHS is a federal discretionary provision program subject to annual Congressional appropriations (21, 165). It provides direct or contracted health services to federally recognized tribal members until funds run out. Therefore, the IHS faces the longstanding challenge of underfunding (166). At the aggregate level, in FY2023, the $49.8 billion IHS proposed budget, requested by the Tribal Budget Formulation Workgroup consisting of tribal leaders nationwide, was cut by approximately 80% to the $9.3 billion President proposed budget and ultimately to the $6.9 billion Congressional appropriation (167). At the per capita level, in 2005, Congress appropriated $2,130 for IHS serving AIANs, nearly half of $4,000 for the Bureau of Prisons serving prisoners (168), and lower than $5,000 for Medicare, $5,200 for Veterans Administration (VA), and $7,600 for Medicaid (168). In 2016, federal spending was continuously little, only $2,834 on IHS, lower than $7,492 on Medicaid, $9,404 on VA, and $12,744 on Medicare, according to the US Commission on Civil Rights (12, 169).

The underfunding problem has originated from federal budget planning and approving stages and led to understaffing and under-servicing consequences, such as delaying and forgoing care (170–172). Policies must examine the rationalities of the President, House, and Senate to cut IHS budgets. The Senate Committee on Indian Affairs may request that the GAO investigate the IHS underfunding problem to better fulfill the legal obligation of the federal government as trustee of tribal trust land.

#### 7.1.4 FDA pre-market vaccine clinical trials lack AIAN representativeness

Clinical trials (e.g., Gardasil 9) usually randomly sample the general population, thus targeting virus genotypes (e.g., HPV-6 and 11) most prevalent in that population, while not oversampling AIANs, thus not targeting genotypes (e.g., HPV-51) most prevalent among AIANs (173–175). The FDA may require clinical trials to oversample the AIAN population.

#### 7.1.5 CDC and FDA post-market vaccine safety surveillance lack AIAN representativeness

Three US post-market surveillance systems lack AIAN representativeness, whether self-reported (176), provider-reported (177), or EHR-based Vaccine Safety Datalink, VSD (178). The VSD consists of no I/T/U facilities at primary sites in California, Colorado, and Georgia, none sufficiently populated by AIANs. The National Indian Health Board recommends to: (1) improve federal, state, and tribal partnerships to increase AIAN representation in data collection; (2) increase data funding for I/T/U facilities and IHS-Tribal Epidemiology Centers (TECs); (3) increase funding for recruiting and training tribal public health specialists (e.g., epidemiologists, statisticians, informaticians); (4) increase recognition of tribal data sovereignty (179).

### 7.2 Tribal-level factors

#### 7.2.1 Tribal sovereignty, self-governance, and self-determination

Tribal sovereignty includes the “right to establish their own government, determine membership requirements, enact legislation and establish law enforcement and court systems,” as well as healthcare systems (90). Under Public Law 93–638, tribes can administer services the IHS would otherwise provide through two ways (90): (1) Title I Self-Determination Contracting, where tribes contract with the IHS to administer individual programs; (2) Title V Self-Governance Compacting, where tribes assume full funding and control over programs (180). Either way allows tribes to address their specific health needs. During the early COVID-19 vaccine rollout till September 2021, AIANs had the highest full vaccination rate of 47.5% among all racial groups (67). Besides timely vaccine distribution (section 5.2.2), such success was attributed to tribal self-determination in allocating and administering vaccines. First, allocation prioritized members critical to tribal culture, language, and health. Seattle Indian Health Board defined the elder age as 50 rather than 65 years because of AIAN’s chronic health disparities (67, 81, 90). Second, tribes generally used financial incentives, such as cash, lottery, or free play at tribal casinos (181). Policies may support tribes in addressing their specific health needs and expanding successful self-determination in COVID-19 vaccine allocation and administration to routine and future vaccines.

#### 7.2.2 School immunization requirements for AIAN children

Requirements depend on the type of school. There are 183 BIE-funded elementary and secondary schools, including 53 BIE-operated and 130 tribally-operated, on 64 reservations in 23 states, serving about 46,000 AIAN students (182). Only BIE-operated school employees, not students, were required to get COVID-19 vaccine (183, 184). Other charter schools are state-funded and subject to state mandates with exemptions (185, 186). No mandates by tribal governments were found. BIE-funded schools may educate students and parents on routine vaccine benefits, including preventions of school and work sick leaves, associated education and wage losses, and fatal childhood VPDs. In addition, each tribal government may refer to CDC-recommended routine vaccines and state mandates, assess its specific health needs (prevalence of VPDs) using IHS-TECs data, and recommend or mandate needed vaccines to its members. Tribal governments may further consider discussing potential recommendations or mandates and shaping a standard policy at the Health Subcommittee of Human Resources Committee at the National Congress of American Indians.

### 7.3 State-level factor

Medicaid is co-financed by federal and state governments. It subsidizes immunization for low-income AIAN enrollees, regardless of federal recognition status (Section 7.1.1).

### 7.4 Manufacturers undersupply vaccines for virus genotypes prevalent among AIANs

Lacking AIAN representativeness in clinical trials led to lacking estimates of virus genotypes that are highly prevalent among AIANs, thus undersupplying certain vaccines for AIANs (section 7.1.4) HPV-51 prevalence rates among AIAN women were 4.9% in the Hopi Tribe of Arizona and 7.6% in a tribe in the Great Plains, higher than the 1.5% national average (176). DHHS agencies may coordinate policies regarding data, research, and development. IHS may request its CDC-partnered TEC Public Health Infrastructure (187) for surveillance data on VPDs. NIH may then fund research to use surveillance data to identify VPDs disproportionally prevalent among AIANs. FDA may then release a list of rare VPDs and incentivize manufacturers to develop vaccines as orphan drugs. For example, a 4.9–7.6% prevalence rate of AIAN women, as 1.1% of the 331 million US population (188), would result in 179,000–277,000 potential infections. If less than 200,000, HPV-51 would meet the definition of an orphan disease and qualify for manufacturer incentives, including clinical trial tax credits, FDA regulatory fee exemption, and post-FDA approval market exclusivity (189).

### 7.5 Researchers lack access to restricted data on AIAN race

AIAN race data are restricted in most CDC national surveys (190). Access to data requires time-and money-consuming applications and travels to only a few designated locations without digital access (191). Missing or masking the AIAN racial category as “other” can obscure AIANs’ disproportionate VPD burdens, thus their specific health needs for government resource allocations (192–194). Data collection and reporting should follow the US Office of Management and Budget racial classification standards, with AIAN as a distinct category (192, 195). CDC should reduce researchers’ time and financial barriers to access AIAN race data.

### 7.6 Structural racism leads to AIANs’ low overall vaccination coverage

Structural racism refers to structures that continue unfairly treating people based on race (196–198). Trust land laws, such as the Indian Removal Act of 1830 (199), the Indian Appropriations Act of 1871 (200), and the General Allotment Act of 1887 (121), have led to AIANs’ lost self-sufficient economy and identity, as well as public health issues (90).

Treaties promised tribes federal funds for limited health resources, such as a physician each for the Kiowa, Comanche, and Makah Tribes who “shall furnish medicine to the sick and shall vaccinate them” (90). However, the limited resources (201) no longer meet the health needs of the 3.7 million AIAN population nowadays (139).

The Infrastructure Investment and Jobs Act of 2021 appropriated about $10 billion to the DOI to address environmental issues, invest in tribes, and create jobs (202, 203). As of April 2024, the DOI spent only $0.26 billion on tribal investments nationwide and $0 in Oklahoma, the original “Indian Territory” with one of the largest AIAN populations (204). Almost all investments were in tribal natural resources and little in human capital and jobs (204), unable to address unemployment and employer-sponsored health insurance challenges among AIANs (205, 206).

Therefore, negative consequences of structural racism exist on AIANs as health service consumers and on health providers and insurance for AIANs; all exacerbate AIAN racial disparities in healthcare consumption and health. Policies must address structural racism. First, Congress must increase IHS appropriations to meet AIAN health needs. Second, the DOI must increase investments in tribal human capital, jobs, and economic development. Third, Title VII of the Civil Rights Act protects employees and job applicants from employment discrimination based on race. The US Equal Employment Opportunity Commission, which enforces the law (207), should investigate discrimination charges brought by AIANs against employers. The US Commission on Civil Rights, which enhances the law enforcement (208), should investigate allegations relating to deprivations because of AIAN race (209).

### 7.7 Comparison of vaccination determinants of AIANs with other racial and ethnic groups

Vaccine hesitancy is a common public health challenge among racial and ethnic minority groups (44, 210, 211). At the Individual level, knowledge from education and health literacy (210, 211), beliefs and preferences (210, 211), insurance status (210), and vaccination cost (211) were common determinants among AIAN and other racial and ethnic minority groups, with higher disparities in Hispanic and African Americans compared to Whites. African Americans have additional determinants, such as culturally-specific home remedies (210, 211), similar to the traditional medicine (26) among AIANs. Common interpersonal-level determinants encompass provider vaccine recommendations. Common organizational-level determinants include the supply of vaccines and vaccination services (212). Common community-level determinants include the geographic location of communities, such as rural residence, the demographic composition of communities served by healthcare organizations, and cultural and linguistic factors (213). Common social-level determinants include structural racism and distrust in governments and health systems. However, Native and African Americans differ in the historical reasons that have led to structural racism and distrust.

## 8 Conclusion

The AIAN population is historically disadvantaged. This article is the first that overviews the unique multi-level determinants and mechanisms of vaccination behavior of the AIAN population from a multidisciplinary perspective. It applies rigorous economic definitions and theoretical model of individual decision-making under uncertainty to understand the decision-making process toward vaccination behavior. Building on individual-level determinants, it applies the social-ecological conceptual framework to identify interpersonal-, organizational-, community-, and societal-level determinants that influence AIAN vaccination. It provides potential mechanisms behind determinants. Its findings highlight the gaps and limitations of laws and policies impacting AIAN vaccination. Correspondingly, it provides future research directions, local interventions, and broad government policies to close the gap and enhance the AIAN vaccination coverage, population health, and healing.

Tribal community interventions may (1) increase AIANs’ knowledge about VPDs, vaccines, and Medicaid enrollment; (2) design risk/cost–benefit calculations using scientific objective probabilities of vaccine safety and efficacy; (3) tailor messages to epidemic histories, narratives, and values; and (4) outreach by trusted messengers. I/T/U organizational interventions may reduce transportation costs while increasing provider supplies, cultural competency, and vaccine standing orders. Federal policies may increase IHS funding, tribal infrastructure, and AIAN data representativeness while eliminating structural racism and generational trauma.
